# A Pilot Study of Wet Lung Using Lung Ultrasound Surface Wave Elastography in an Ex Vivo Swine Lung Model

**DOI:** 10.3390/app9183923

**Published:** 2019-09-19

**Authors:** Xiaoming Zhang, Boran Zhou, Alex X. Zhang

**Affiliations:** 1 Department of Radiology, Mayo Clinic, Rochester, MN 55905, USA;; 2 Department of Biochemistry and Molecular Biology, Mayo Clinic, Rochester, MN 55905, USA;

**Keywords:** lung ultrasound surface wave elastography (LUSWE), lung water, surface wave speed, ex vivo swine lung

## Abstract

Extravascular lung water (EVLW) is a basic symptom of congestive heart failure and other conditions. Computed tomography (CT) is standard to assess EVLW, but it requires ionizing radiation and radiology facilities. Lung ultrasound reverberation artifacts called B-lines have been used to assess EVLW. However, B-line artifact analysis relies on visual interpretation and subjects to inter-observer variability. We developed lung ultrasound surface wave elastography (LUSWE) to measure lung surface wave speed. This research aims to develop LUSWE to measure the change of lung surface wave speed due to lung water in an ex vivo swine lung model. The surface wave speeds of a fresh ex vivo swine lung were measured at four frequencies of 100 Hz, 200 Hz, 300 Hz, and 400 Hz. An amount of water was then filled into the lung through its trachea. Ultrasound imaging was used to guide the water filling until significant changes were visible on the imaging. The lung surface wave speeds were measured. An additional 120 ml of water was then filled into the lung. The lung surface wave speeds were then measured again. The results demonstrated that the lung surface wave speed decreased with respect to water content.

## 1. Introduction

Extravascular lung water (EVLW), or pulmonary edema, is a basic symptom of congestive heart failure and other inflammatory conditions, such as acute respiratory distress syndrome [1]. EVLW assessment is challenging. Chest x-ray or chest computed tomography (CT) are standard to assess EVLW, but these imaging studies pose a significant logistic burden and require ionizing radiation and radiology facilities. Lung ultrasound reverberation artifacts called B-lines have been used to assess EVLW. However, B-line artifact analysis relies on visual interpretation and is subject to inter-observer variability [2].

We developed lung ultrasound surface wave elastography (LUSWE) [3–5] to measure surface wave speed of lung for assessing patients with interstitial lung disease (ILD) [6, 7]. This research aims to develop LUSWE to accurately detect changes in lung stiffness (compliance) caused by EVLW (pulmonary edema). We studied a lung phantom sponge model using LUSWE [8–10]. The purpose of this study was to test LUSWE on *ex vivo* swine lungs.

## 2. Materials and Methods

In ultrasound surface wave elastography (USWE), a 0.1s harmonic vibration at a frequency is generated on the surface of tissue using a small vibrator. The surface wave propagation on the tissue is measured using an ultrasound probe. The 0.1-second harmonic vibration signal is generated by a function generator (Model 33120A, Agilent Inc., Santa Clara, CA). The signal is amplified using a power amplifier (Model PYLE PRO PCA4, PYLE PRO Service Center, 1600 63^rd^ Street, Brooklyn, NY 11204). The signal is then sent to a small vibrator shaker (Model: FG-142, Labworks Inc., Costa Mesa, CA 92626, USA). The shaker generates a local small vibration on the surface of tissue using an indenter with a plastic ball with 5 mm diameter.

A Verasonics ultrasound system (Verasonics, Inc., Kirkland, WA 98034, USA) with an ultrasound probe of L11–5 with a central frequency of 6.4 MHz is used to measure the wave propagation of tissue. USWE can measure both surface wave propagation on the tissue and the shear wave propagation inside the tissue. The radio-frequency (RF) data of ultrasound echo from the tissues are obtained. By demodulation of the RF data using quadrature detection, the in-phase/quadrature (IQ) data of ultrasound signals are processed. Particle velocity *v* due to the external vibration generation at a pixel in the axial direction of ultrasound beam is analyzed. *v* is calculated from the IQ data of consecutive frames using a one-dimensional autocorrelation method [11, 12]. The speed of sound in tissue is assumed to be 1540 m/s. High frame imaging is obtained by using the plane-wave transmission technique. Pulse repetition frequency (PRF) is 2000 pulses per second. Central frequency (*f*) is 6.4 MHz. 3 pixels in the axial direction and 2 sampling points in the slow time direction are used for averaging. Prior to window selection, the whole autocorrelation matrix for each lateral direction is calculated as follows:

(1) $$v=\;\frac{c}{4\pi fT_{s}}\left\{\frac{\sum_{m=0}^{M-1}\sum_{n=0}^{N-2}\left[Q\left(m,n\right)I\left(m,n+1\right)-I\left(m,n\right)Q\left(m,n+1\right)\right]}{\sum_{m=0}^{M-1}\sum_{n=0}^{N-2}\left[I\left(m,n\right)I\left(m,n+1\right)+Q\left(m,n\right)Q\left(m,n+1\right)\right]}\right\},$$

where $T_{s}=\frac{1}{PRF}$ is the pulse repetition period, *I*(*m*, *n*) and *Q*(*m*, *n*) are the real and imaginary IQ date at a pixel (m, n). A 3 × 3 pixel spatial median-filter is then used on each frame of the wave motion image to remove noise spike points.

In the application of this technique for the lung, we measure the surface wave propagation along the lung. The phase change of surface wave propagation over the two locations can be calculated with a cross-spectrum method. *v*_1_(*t*) and *v*_2_(*t*) represent the tissue motion at two locations on the lung surface. The cross-spectrum *S*(*f*) of two signals *v*_1_(*t*) and *v*_2_(*t*) is defined as [13],

(2) $$S(f)=S_{1}^{*}(f)\cdot S_{2}(f)=\left|S_{1}(f)\cdot S_{2}(f)\right|\cdot e^{-j\Delta \varphi (f)},$$

where *S*_1_(*f*) and *S*_2_(*f*) are the Fourier transforms of *v*_1_(*t*) and *v*_2_(*t*), respectively; * denotes the complex conjugate; and Δ*ϕ* (*f*) is the phase change between *v*_1_(*t*) and *v*_2_(*t*) over distance at frequency *f*.

The phase change of surface wave with distance is used to measure the surface wave speed,

(3) $$c_{s}=2\pi f\left|\Delta r/\Delta \phi \right|,$$

where Δ*r* is the radial distance of two measuring locations, Δ*ϕ* is the wave phase change over distance, and *f* is the frequency. The estimation of surface wave speed can be improved by measuring the phase change over multiple locations using a regression model, $\Delta \hat{\phi }=-\alpha \Delta r+\beta$, where $\Delta \hat{\phi }$ denotes the regression value of multiple Δ*ϕ* measurements, *α* and *β* are regression parameters, and *c*_*s*_ (*w*) = *w*/*α*.

**Figure 1.**
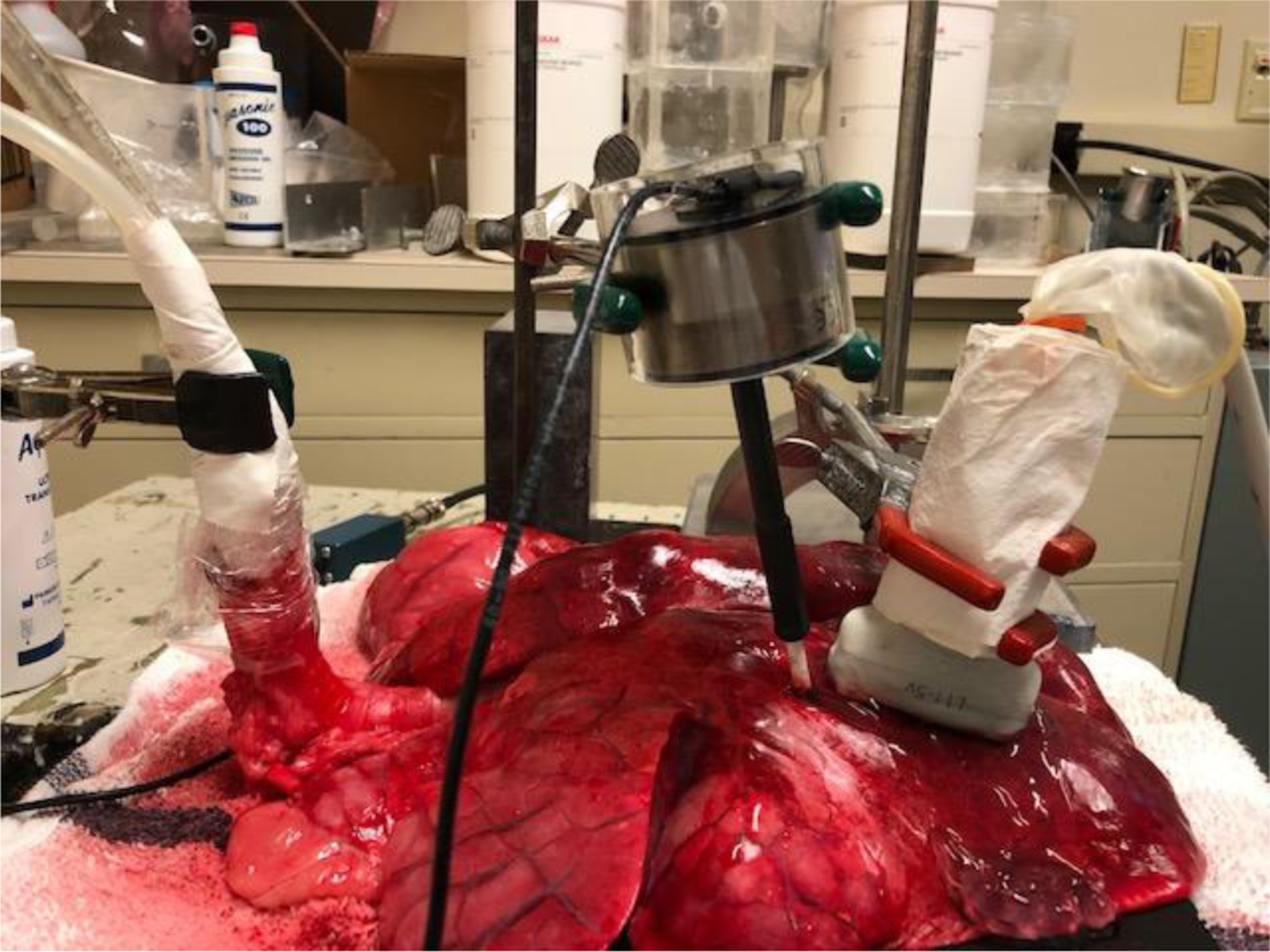
Experimental design for testing the surface wave speed on an ex vivo fresh swine lung. The swine lung was positioned on a testing table. A thick rubber material plate was between the lung and the table to reduce wave reflection. A tube was connected to the trachea of lung to inject the water to the lung. In this ex vivo swine lung study, a 0.1s harmonic vibration at a frequency is generated on the surface of lung using a small vibrator. The surface wave propagation on the lung is measured using an ultrasound probe.

**Figure 2.**
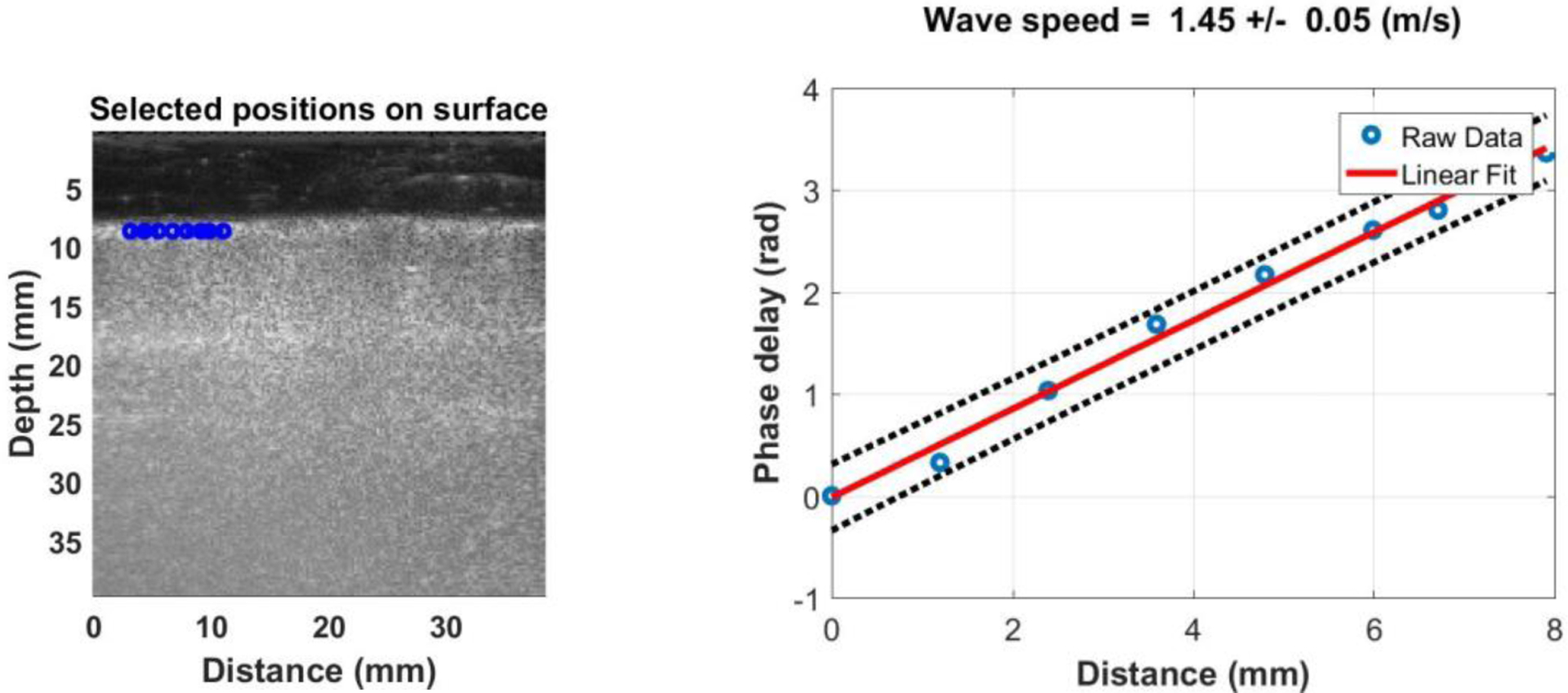
(a) Representative B-mode image of lung at the right lobe of the swine. Eight locations on the lung surface were used to measure the lung surface wave speed. (b) Measurement of lung surface wave speed at 100 Hz. The lung surface mtion at the first location is used as the reference. The wave phase delay of the remaining locations, relative to the first location, is used to measure the lung surface wave speed. Representative examples of wave speed at 100 Hz for the lung.

Figure 1 shows the experimental design for testing the surface wave speed on a fresh *ex vivo* swine lung. The swine lung was positioned on a testing table. A thick rubber material plate was between the lung and the table to reduce wave reflection. A tube was connected to the trachea of lung to inject the water to the lung. Figure 2a shows a representative B-mode image of the lung. Data of the lung were acquired by compounding 11 successive angles at a pulse repetition frequency (PRF) of 2 kHz. On the lung surface, eight locations were used to measure the surface wave speed of lung. The surface motion velocity was in response to the external vibration excitation induced by the shaker. Using the lung motion at the first location as a reference, the wave phase delay of the lung motions at the remaining locations—relative to the first location—was used to measure the surface wave speed. Figure 2b shows a representative wave speed at 100 Hz for the lung. The surface wave speed was 1.45 ± 0.05 m/s for the lung. The lung was tested at both the right and left lobes. The surface wave speed was measured at four excitation frequencies of 100 Hz, 200 Hz, 300 Hz and 400 Hz. Five measurements were performed for each lung side and each frequency. The surface wave speed was analyzed in the format of mean ± SD for the five measurements at each side and each frequency. A small tissue motion in tens of μm was enough for sensitive ultrasound detection of the generated tissue motion. The 100 Hz excitation signal is stronger than those of higher frequency excitations. The higher frequency waves have smaller wave length but decay more rapidly over distance than the lower frequency waves. The frequency ranges chosen in this study consider the wave motion amplitude, spatial resolution, and wave attenuation.

## 3. Results

**Figure 3.**
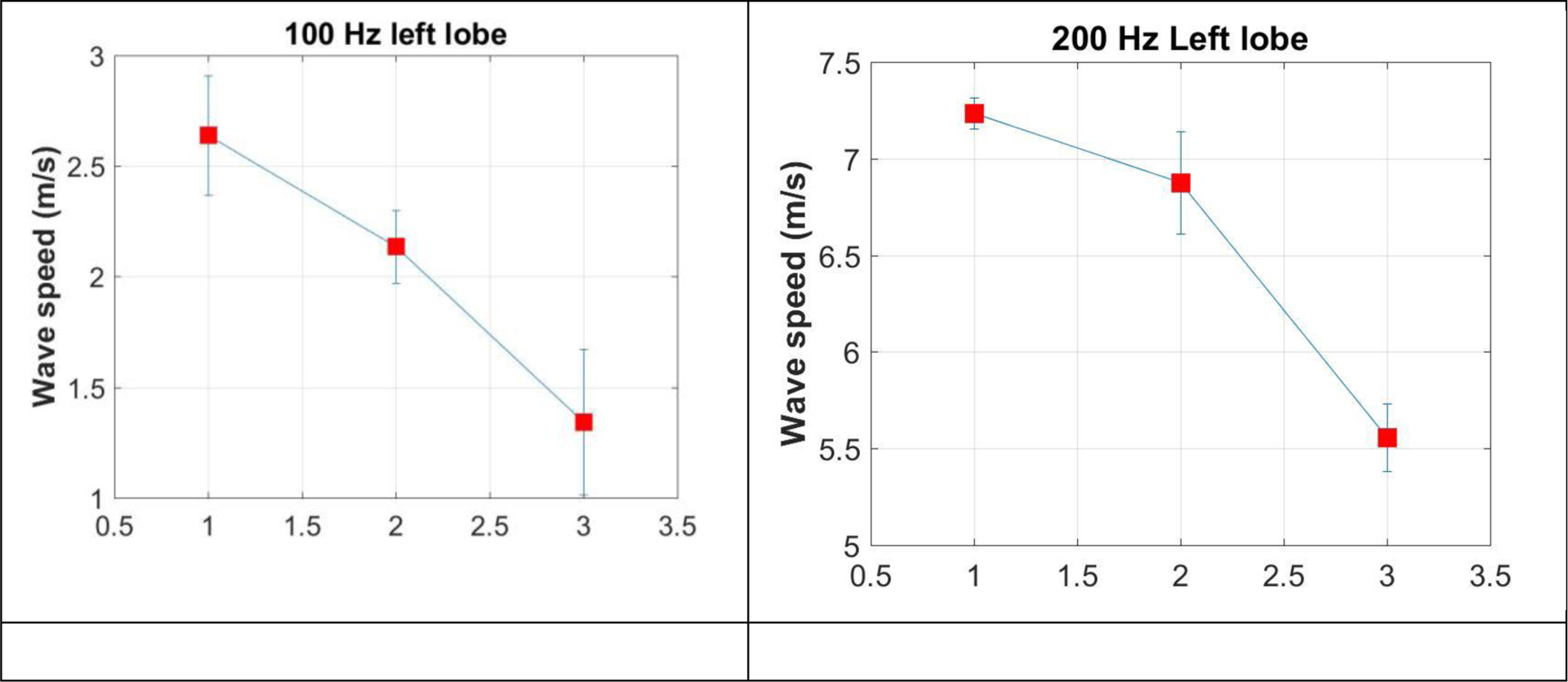

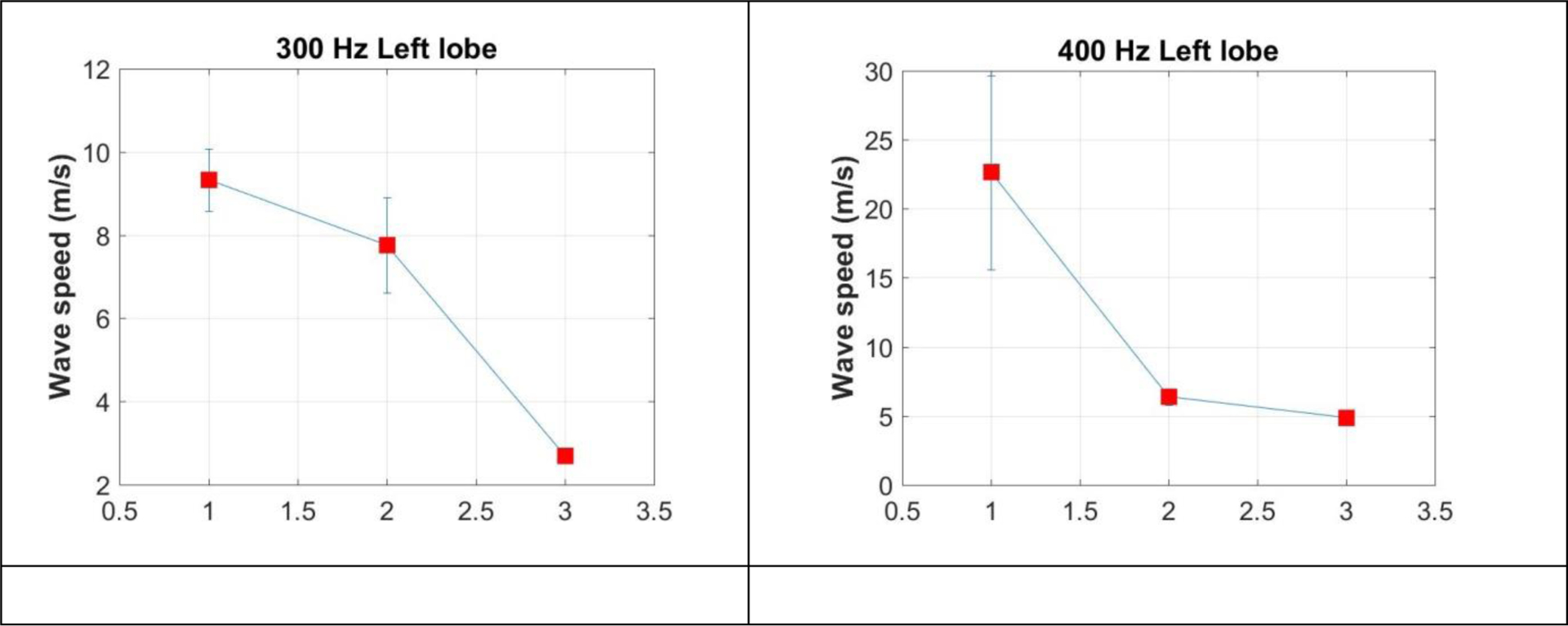
The surface wave speeds at the left lobe of lung at the baseline, water injection, and additional 120 ml water injection for 100 Hz (a), 200 Hz (b) 300 Hz (c), and 400 Hz (d). The figure x coordinate represents the baseline, water injection, and additional 120 ml water injection with values of 1, 2, and 3.

**Figure 4.**
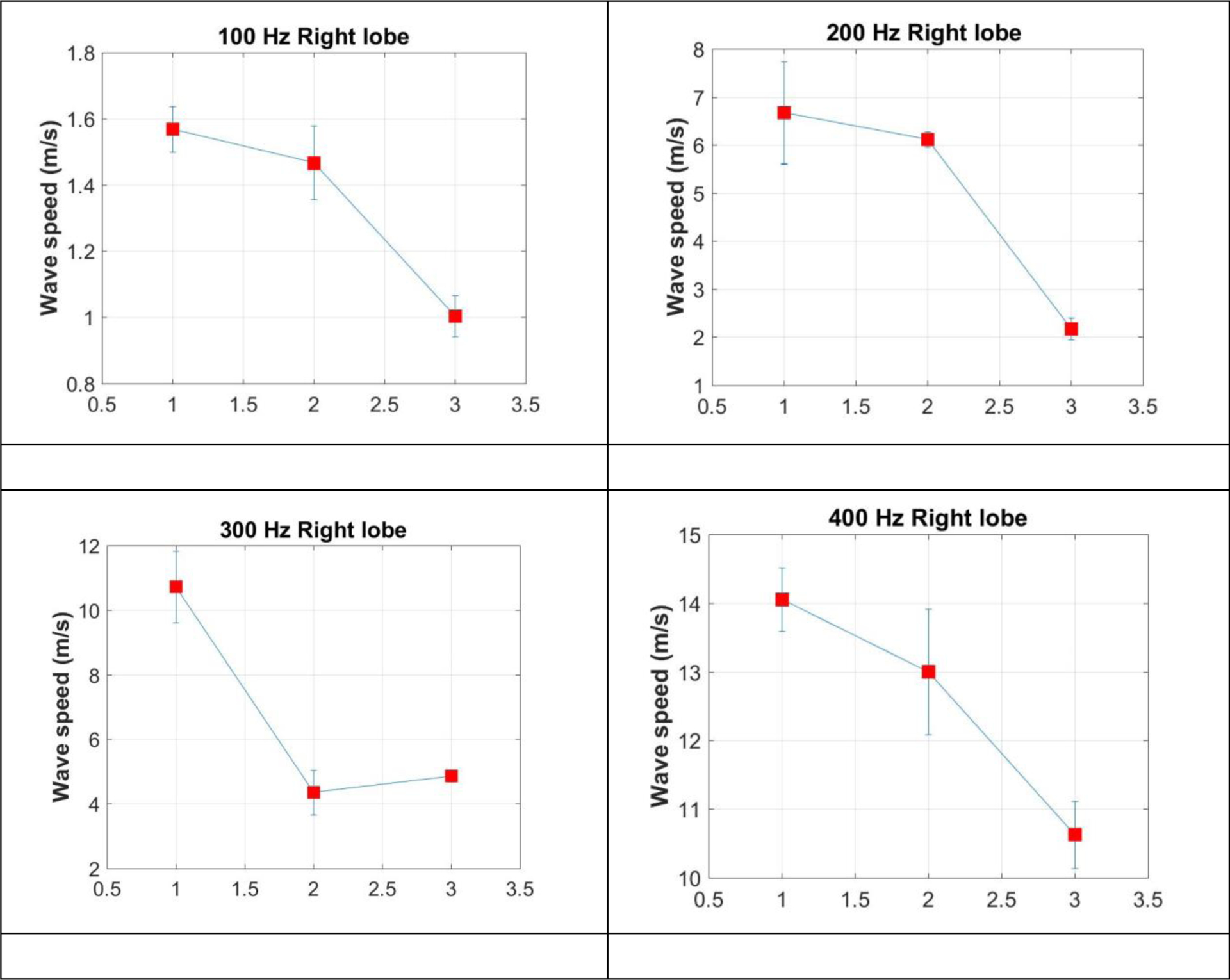
The surface wave speeds at the right lobe of lung at the baseline, water injection, and additional 120 ml water injection for 100 Hz (a), 200 Hz (b) 300 Hz (c), and 400 Hz (d).

The lung was tested at the baseline and at one lobe of the lung first. Five measurements were performed for the four frequencies of 100, 200, 300, and 400 Hz. The measurements were then performed for the other lobe of the lung. After both lobes of the lung were tested, some water was injected to the lung through its trachea. Ultrasound imaging was used to guide the water filling until significant changes were visible on the imaging. The lung surface wave speeds were measured at both lobes of the lung. Then, an additional 120 ml of water was then filled into the lung. The lung surface wave speeds were measured again. Figure 3 shows the surface wave speeds at the left lobe of lung at the baseline, water injection, and additional 120 ml water injection for 100 Hz (a), 200 Hz (b) 300 Hz (c), and 400 Hz (d). Figure 4 shows the surface wave speeds at the right lobe of lung at the baseline, water injection, and additional 120 ml water injection for 100 Hz (a), 200 Hz (b) 300 Hz (c), and 400 Hz (d). The figure x coordinate represents the baseline, water injection, and additional 120 ml water injection with values of 1, 2, and 3.

## 4. Discussion

The aim of this study was to evaluate if the lung surface wave speed was affected by lung water in an *ex vivo* swine lung model. The lung water was injected to the lung through the trachea of lung. Previously, we studied the change of lung surface wave speed with pulmonary pressure [14]. The pulmonary pressure was controlled by pumping the air through the trachea of lung. The pulmonary pressure was monitored using a pressure sensor. In this pilot study on lung water, the lung was tested at the baseline. Then some water was injected to the lung through its trachea. Ultrasound imaging was used to guide the water filling until significant changes were visible on the imaging. The lung surface wave speeds were measured. An additional 120 ml of water was then filled into the lung. The lung surface wave speeds were measured again. For future research, the quantification of water volume needs to be improved. In this study we quantified the water volume based on injection volume. Another viable method of quantifying water volume is measuring the water pressure of the lung

A high pulse repetition rate of 2000 frame/s was used to detect surface lung motion in response to the excitations of 100 Hz, 200 Hz, 300 Hz and 400 Hz. A Verasonics ultrasound system was used to collect up to 2 thousand imaging frames per second by using a plane-wave pulse transmission method. The surface lung motion velocities at these locations were measured in the normal direction of the lung using the ultrasound tracking beams through those locations [14, 15]. The lung surface wave speed was measured by analyzing ultrasound data directly from the lung. Therefore, the wave speed measurement was local and independent of the location of excitation. The lung surface wave speeds of both left and right lung lobes were tested at four excitation frequencies. Viscoelasticity of the lung can be analyzed using the wave speed dispersion curve if the lung mass density is known. Further studies on lung mass density will be performed.

We can identify the decrease of lung surface wave speed with lung water for both lobes of the lung and for the four frequencies. At the left lung and at 200 Hz, the surface wave speed increased a little with water injection but decreased with additional water injection. This is an interesting phenomenon. We could not identify the trend of wave speed with water in the sponge phantom model [9]. In the sponge phantom model, the change of mass density of phantom could be measured. Using the viscoelastic model, we can identify that the shear viscosity of the sponge increased with water content and shear elasticity exhibited a subtle increase. We will try to quantify the lung mass density in *ex vivo* swine lungs so that we can estimate viscoelasticity of lung based on the wave speed measurements at the four frequencies.

## 5. Conclusions

LUSWE is a noninvasive technique for measuring the surface wave speed of lung. In this study, an ex vivo swine lung model was evaluated to measure the change of lung surface wave speed due to lung water. An ex vivo fresh swine lung were tested at both the right and left lobes of the lung. The lung surface wave speeds were measured at four frequencies of 100 Hz, 200 Hz, 300 Hz, and 400 Hz. Then an amount of water was filled into the lung through its trachea. Ultrasound imaging was used to guide the water filling until significant changes were visible on the imaging. The lung surface wave speeds were measured. An additional 120 ml of water was then filled into the lung. The lung surface wave speeds were measured again. The results demonstrated that the lung surface wave speed decreased with respect to water content.
